# Influence of ejaculation frequency on seminal parameters

**DOI:** 10.1186/s12958-015-0045-9

**Published:** 2015-05-21

**Authors:** B. Jose Manuel Mayorga-Torres, Mauricio Camargo, Ashok Agarwal, Stefan S. du Plessis, Ángela P. Cadavid, Walter D. Cardona Maya

**Affiliations:** Grupo Reproducción, Departamento de Microbiología y Parasitología, Facultad de Medicina, Sede de Investigación Universitaria, Universidad de Antioquia, Calle 70 No. 52-21, Medellín, Colombia; Grupo Genética, Regeneración y Cáncer, Facultad de Ciencias Exactas y Naturales, Universidad de Antioquia, Medellín, Antioquia Colombia; Center for Reproductive Medicine, Cleveland Clinic, Cleveland, OH USA; Division of Medical Physiology, Faculty of Medicine and Health Sciences, Stellenbosch University, Tygerberg, South Africa

**Keywords:** Sperm quality, Ejaculation frequency, Seminal parameters, Reactive oxygen species, DNA fragmentation, Mitochondrial membrane potential, Flow cytometry analysis

## Abstract

**Background:**

Several factors have been shown to influence semen parameters, one of which is sexual abstinence; a clinical criteria included in the semen evaluation to provide maximum sperm quality. The aim of the present study was to assess the effect of a daily ejaculation frequency on conventional and functional semen parameters.

**Methods:**

Semen samples were collected daily over a period of two weeks of which every second sample per person was processed and analyzed according to the World Health Organization guidelines. Furthermore, mitochondrial function, intracellular reactive oxygen species production and sperm DNA fragmentation were evaluated by flow cytometry.

**Results:**

Total sperm count and seminal volume per ejaculation declined and remained decreased for the duration of the daily ejaculation period. However, conventional parameters such as sperm concentration, motility, progressive motility, morphology, vitality and functional parameters such as sperm plasma membrane integrity, mitochondrial membrane potential and DNA fragmentation was not significantly affected and remained similar to the initial measurement throughout the daily ejaculation period. Despite intra- and inter individual variations, the average values of the basic semen parameters remained above the WHO (2010) reference values throughout the daily ejaculation period. Interestingly, a decreasing trend in intracellular ROS production was observed, although statistically not significant.

**Conclusions:**

The study shows that an extended 2 week period of daily ejaculation does not have major clinical effects on conventional and functional seminal parameters.

## Background

Once human spermatozoa have been produced in the seminiferous tubuli, they are stored in the epididymis for future release [1]. Unlike other species, the male gamete of mammals must pass through the epididymis, where they undergo a series of physiological and biochemical changes, allowing them to mature and acquire fertilizing potential [1]. Epididymal transit time has been estimated to range from 2 to 11 days [2]. The variation is probably due to the rate of passage through the cauda which in turn can be influenced by ejaculatory frequency [3, 4]. The negative effect of prolonged storage (epididymal transit) on sperm motility has been reported widely, but the effective storage period in the human is still uncertain [3, 5]. More recently new insights have been gained through the research conducted on epididymal function and its regulation of spermatozoa [6–8].

Several factors have been shown to influence semen parameters, one of which is sexual abstinence; a clinical criteria included in the semen evaluation to provide maximum sperm quality. The World Health Organization (WHO) guidelines recommend an abstinence period of 2–7 days prior to semen collection for standard seminal evaluation [9]. However, the European Society of Human Reproduction and Embryology (ESHRE) and the Nordic Association for Andrology [10], suggest tighter abstinence intervals of between only 3–4 days. The impact of sexual abstinence on conventional sperm parameters is still controversial [11, 12]. There is general agreement that semen volume and sperm concentration will increase with prolonged sexual abstinence, but simultaneously it can have a negative impact on sperm motility and viability [13–15]. Some other authors have suggested that spermatozoa are greatly exposed to reactive oxygen and nitrogen species (ROS and RNS) during epididymal maturation and storage [16, 17]. Spermatozoa are extremely susceptible to oxidative attack and this has been well-correlated with decreased sperm motility, lipid peroxidation, DNA damage and impaired fertilization rates [18–21].

The search for a cost effective fertility treatment has been important to optimize the likelihood of achieving pregnancy. It has been proposed that recurrent ejaculations [22] could be an approach to improve sperm DNA quality and therefore reproductive outcome [11, 12]. The aim of the present study was to assess the effect of daily ejaculation (DE) for 2 consecutive weeks on conventional semen parameters as well as the physiological sperm characteristics such as mitochondrial function, intracellular ROS production and sperm DNA fragmentation index (DFI) as further indicators of sperm production quality and the implication for fertility treatment.

## Materials and methods

Ethical approval for this study was obtained from the institutional Research Ethics Committee, and all men provided informed consent. This study used semen samples collected between May and July 2014. Six healthy men (26.7 ± 4.8 years) were recruited from men serving as quality controls for studies at the Reproduction Group at the Medical School of the University of Antioquia.

Exclusion criteria for study participation were any history of urogenital surgery, leukocytospermia (white blood cells > 1 × 10^6^ cells/mL semen), and azoospermia. In addition, self-reported illnesses or use of medication in the three month immediately preceding the study.

### Semen analysis

Semen samples were produced by masturbation, collected into sterile sample containers and delivered to the laboratory within 1 h of ejaculation. For the first evaluation, a 3–4 day period of sexual abstinence was required and all samples were classified as normozoospermic. Thereafter participants ejaculate once per day for a period of 2 weeks (DE period) during which samples from alternative days (i.e. days 2, 4, 6, 9, 11, and 13) were analyzed for conventional and functional parameters according to the WHO 2010 guidelines [9]. The volunteers did not take any medications and were sexually inactive during the two week DE period. Sperm morphology was analyzed according to the Tygerberg Strict Criteria [23], while the concentration was determined using a Makler chamber (Sefi-Medical Instruments, Haifa, Israel) [24].

### Intracellular ROS production

The intracellular sperm ROS (H_2_O_2_, HO^−^, ONOO^−^) levels were evaluated by flow cytometry using 2’,7’-dichlorofluorescein diacetate (DCFH-DA; Sigma-Aldrich, St Louis, MO), a cell-permeable probe that is highly sensitive to cellular oxidation and fluoresces when oxidized to DCF. It is therefore useful for the detection of ROS and nitric oxide (NO) and for the determination of the degree of overall oxidative stress [25]. Propidium iodide (PI) (Molecular Probes Inc, The Netherlands) was used in conjunction with DCFH-DA as a vitality stain (final concentration 12 μM). DCFH-DA was diluted to a final concentration of 1 μM in Ham´s F12 medium containing 2 × 10^6^ spermatozoa in 300 μL. The cell suspensions were then incubated for 10 min at 37 °C before being analyzed using a flow cytometer. Results are expressed as the mean fluorescence intensity (MFI) of live cells exhibiting a fluorescent response.

### Sperm chromatin structure assay

The Sperm Chromatin Structure Assay (SCSA) was used to measure the DNA fragmentation index (DFI), as previously described by Evenson et al. [26, 27] with some modifications developed in our laboratory [28]. The sperm suspension (200 μl) was added to a cytometry tube containing 200 μl of acid detergent solution. After 30 s, the spermatozoa were stained with 600 μl of acridine orange staining solution to give a final concentration of 6 μg/mL. The ratio of single stranded (red) to single plus double stranded (green) fluorescence were expressed as the %DFI.

### Mitochondrial membrane potential

Mitochondrial membrane potential (MMP) were measured by flow cytometry using 3,3′-dihexyloxacarbocyanine iodide (DIOC6; Molecular Probes Inc, The Netherlands) staining. DIOC6 is a cell-permeant, green-fluorescent cationic lipophilic dye that is selective for the mitochondria of live cells when used at low concentrations. PI was used as a vitality counter stain. Briefly, 2 × 10^6^ spermatozoa suspended in 300 μL of medium were incubated for 20 min at 37 °C with DIOC6 (final concentration of 10nM) and subsequently subjected to flow cytometry. Samples were scored as the percentage cells in the population showing high MMP.

### Plasma membrane integrity evaluation

The integrity of the plasma membrane was assessed with the LIVE/DEAD® Sperm Viability Kit (Molecular Probes Inc, The Netherlands). Staining was carried out according to the manufacturer’s instructions. In brief, 300 μl of sperm suspension, containing 2 × 10^6^ spermatozoa was incubated with SYBR-14 and PI to final concentrations of 1 μM and 12 μM respectively prior to flow cytometry analysis. Data are expressed as the percentage of viable sperm positive to SYBR-14 and negative to PI. All flow cytometry analyses reported in this study were conducted on an Epics XL flow cytometer (Becton Dickinson, CA, USA) with a 488 nm argon laser. Forward scatter and side scatter measurements were taken to generate a density plot, which was used to gate for sperm cells only. All data were acquired and analyzed using WinMDI 2.9 Software (Scripps Research Institute, La Jolla, CA) and a total of 10,000 events were collected per sample.

### Statistical analysis

To compare the variables between groups without assuming that values follow a Gaussian distribution the non-parametric Mann–Whitney test was used. Data were analyzed using Prism 5.0 (GraphPad Software, San Diego, CA) statistical software and a p value of <0.05 was considered to be statistically significant. All data are expressed as the mean ± standard error of mean (SEM).

## Results

All the semen samples were normozoospermic (concentration > 15 x l0^6^ sperm/mL, progressive motility > 32 %, vitality > 58 %, and normal forms > 4 %) at the first evaluation (after 3–4 days of abstinence).

As expected, seminal volume were reduced during the DE period. Similarly, the total number of spermatozoa per ejaculate was also reduced when compared to the initial evaluation (p <0.05). These observations were already manifested on Day 2. Interestingly, sperm concentration, motility, progressive motility and morphology did not differ significantly from the first evaluation throughout the DE period as can be seen in Table 1. However, a wide intra-individual variation was observed in each of these parameters. The subtle changes in motility and sperm viability are worth noting, with the number of viable spermatozoa actually increasing on day 13 of the experiment compared to the initial evaluation (69.8 ± 5.6 % vs. 60.2 ± 3.1 %, p < 0.05). All of these basic parameters furthermore remained above the 2010 WHO reference values throughout the DE period except for the viability on day 4 (53.7 ± 7.4 %), which decreased to below the 58 % reference value (see Table 1).

**Table 1:** Semen parameters for the first evaluation and during the daily ejaculation period of 2 weeks. (Mean ± SEM, n = 6)

	First	Day 2	Day 4	Day 6	Day 9	Day 11	Day 13
Abstinence, days	3-4	1	1	1	1	1	1
Volume, mL	2.5 ± 0.2	1.8 ± 0.4*	1.8 ± 0.3*	1.6 ± 0.2*	1.9 ± 0.3*	1.7 ± 0.4 *	1.9 ± 0.2*
Sperm concentration, 10^6^/mL	121.3 ± 35.5	80.3 ± 18.7	72.1 ± 18.1	66.1 ± 15.2	75.1 ± 22.6	80.4 ± 21.5	80.3 ± 19.2
Total sperm count, ×10^6^	276.8 ± 70.5	134.4 ± 46.6*	115.7 ± 27.8*	88.7 ± 16.2*	111.5 ± 23.6*	109.9 ± 27.3*	138.2 ± 31.4*
Total motility, %	53.83 ± 4.7	55.50 ± 10.2	54.0 ± 7.8	52.9 ± 7.0	58.5 ± 5.7	58.7 ± 4.4	51.70 ± 5.5
Progressive motility, %	47.0 ± 5.5	51 ± 11.3	43.0 ± 8.6	45.2 ± 8.0	46.6 ± 6.4	50.5 ± 5.0	40.8 ± 3.3
Vitality, %	60.2 ± 3.1	61.7 ± 8.8	53.4 ± 7.4	65.5 ± 5.3	60.2 ± 4.7	59.5 ± 3.1	69.8 ± 5.6*
Morphology, normal forms %	13.8 ± 8.2	12.1 ± 9.3	10.3 ± 7.6	11.5 ± 6.8	10.4 ± 9.4	12.9 ± 9.1	10.2 ± 6.9
ROS production, MFI	84.8 ± 9.3	58.5 ± 1.6	64.3 ± 3.8	79.7 ± 13.7	71.8 ± 18.6	63.3 ± 15.4	42.6 ± 6.1
Mitochondrial Membrane Potential, %	49.4 ± 8.1	49.8 ± 12.1	43.3 ± 9.2	49.5 ± 9.7	48.0 ± 6.0	47.7 ± 7.2	51.5 ± 8.6
DNA fragmentation index, %	25.6 ± 3.0	22.1 ± 4.3	24.0 ± 3.4	27.1 ± 3.5	24.7 ± 2.8	25.8 ± 4.6	23.9 ± 4.7
Membrane integrity, %	59. 9 ± 7.4	51.2 ± 7.5	53.0 ± 5.9	63.0 ± 5.8	56.7 ± 6.9	58.7 ± 4.4	57.8 ± 6.8

*MFI* Mean Fluorescence Intensity, *MMP* Mitochondrial Membrane Potential, *ROS* reactive oxygen species

*p < 0.05 *vs*. first analysis

**Fig. 1:**
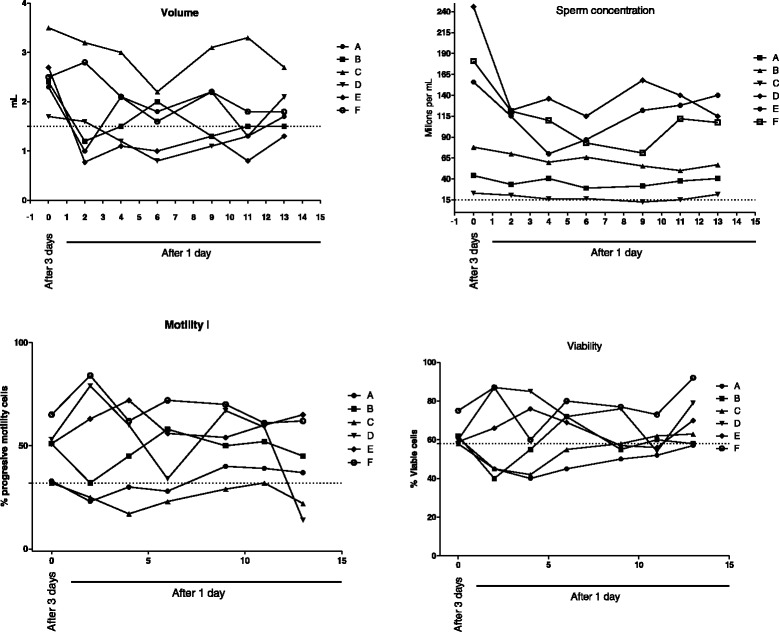
The intra- and inter individual variation of the conventional sperm parameters. Distribution of conventional parameters (semen volume, sperm concentration, motility and viability) during the evaluation period of daily frequent ejaculation. n = 6 individuals (A-F) for each parameter

**Fig. 2:**
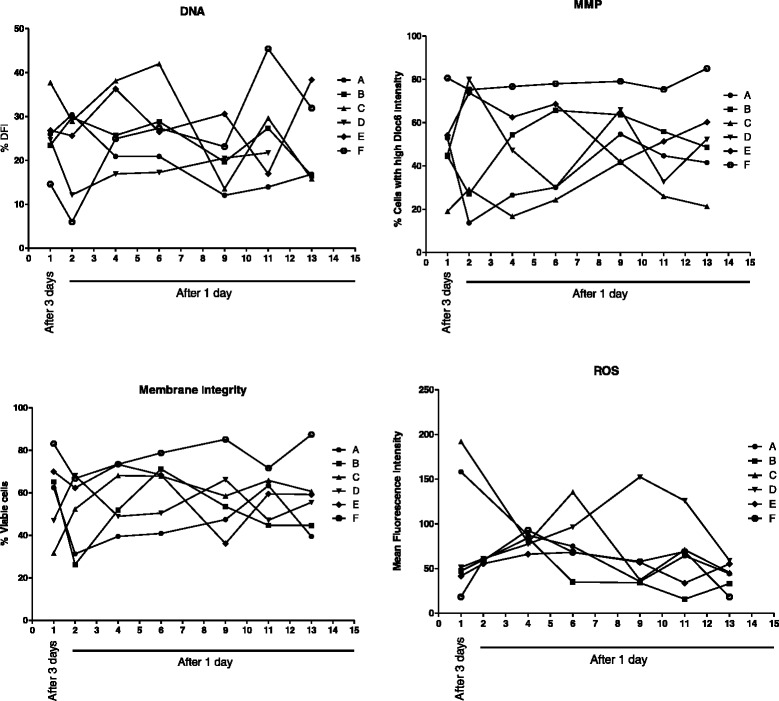
The intra- and inter individual variation of the functional sperm parameters. Distribution of functional parameters (DNA fragmentation index, mitochondrial membrane potential, plasma membrane integrity, reactive oxygen species) during the daily ejaculation period. n = 6 individuals (A-F) for each parameter (DFI: DNA Fragmentation Index, MMP: Mitochondrial Membrane Potential, membrane integrity percent, and the production of intracellular ROS: Reactive Oxygen Species)

None of the functional parameters were significantly influenced by two weeks of DE. The integrity of the sperm plasma membrane (above ≈ 50 % viable cells) and MMP remained stable throughout the period. The percentage of DNA fragmentation did not differ significantly from the initial measurement. DFI remained within acceptable levels (<29 %). With regards to the production of intracellular ROS, a trend of reduction was observed over time (statistically not significant). The intra and inter individual variation of both the conventional and functional seminal parameter over the DE period are depicted in Figs. 1 and 2.

## Discussion

Semen analysis implies the evaluation of several characteristics of the ejaculate with the intent to estimate the reproductive chance/probability of an individual. Studies have found that human semen samples vary over time and this may be due to three principal factors: (i) pre-analytical influences (in the case of semen: sexual abstinence period and transport of the sample to the laboratory); (ii) analytical randomization (precision) and systematic error (bias); and (iii) inherent biological variation [9, 29–31].

The search for predictors of male fertility has resulted in the standardization of procedures for the examination of human semen; an example of this is the guidelines developed by WHO to process seminal samples based on global demographic population studies [9]. This effort has made a solid contribution to semen analysis and has provided a better understanding of human sperm quality.

In the present study, we found that daily ejaculation had an effect on some conventional and functional semen parameters. The main change was already observed after two days of DE, when the seminal volume and the total sperm count were reduced around 70 and 50 % respectively, compared to the first evaluation. This evidence is in accordance to findings from other studies where it was also reported that reduced sexual abstinence had an impact on sperm count and seminal plasma contribution [15, 32].

We did not observe a major change in any of the other conventional sperm parameters such as motility, viability and morphology (Table 1). Throughout the second week of DE (day 9–13), all the conventional semen parameters were maintained except for sperm viability which improved to significantly higher levels on the final day of study (Table 1).

On the other hand, functional parameters including membrane integrity, MMP, and even DNA fragmentation did not change and their values were maintained over the two week DE period (Table 1). However, a decrease, although statistically not significant, in intracellular ROS production was observed as early as the second day of high ejaculation frequency and these levels were maintained well below that of the first evaluation (MFI: 84.8, Table 1). It has been well described in studies of ROS production and sperm physiology that these highly reactive chemical species play a major role in many sperm processes such as maturation, motility and capacitation [33]. Nevertheless, the ROS levels must be controlled within physiological levels as overproduction or lack of sufficient antioxidant systems can lead to the development of oxidative stress. As these compounds are highly reactive they can cause lipid peroxidation, DNA damage and apoptosis which impacts directly on both conventional as well as functional sperm parameters [21].

We observed that during periods of high ejaculation frequency the sperm concentration initially decrease, but then remained within the same range for the remainder of the DE period, similar results have been previously reported [34]. The initial total sperm count was substantially higher and that can be ascribed to the fact that reserves were available and stored in the epididymis. Due to the increased ejaculation frequency the reserves were depleted and total sperm count decreased, these decreased levels of sperm count can be perceived as the daily spermatogenic production. There are several factors that may influence spermatogenesis including metabolic, genetic, environmental and physiological factors. More than likely, the increased daily spermatogenesis, greater epididymal sperm storage and more sperm in the ejaculate is resultant of evolutionary processes due to sperm competition in mammals and some other species [35–38]. Borgerhoff Mulder, on the other hand supports another idea which hypothesize that the human species adopted a reproductive strategy of high-investment and low-fertility during gamete production [39]. The interesting fact is that despite the limited time between ejaculations and lack of accumulating and storing more spermatozoa, the sperm concentration or total count did not drop to below the WHO reference values during a 2 week period of DE.

ROS are ubiquitous in life and death processes of cells and is a key player in intracellular signaling, host defense, cell death and adaptation processes [40, 41]. The reduction in intracellular ROS levels may underline the role of epididymal function and the time spent in the epididymis; some authors have proposed that spermatozoa are greatly exposed to ROS and RNS during epididymal transit and storage [11]. The decrease that was observed in intracellular ROS levels in samples collected after only 1 day of abstinence can possibly be ascribed to the fact that these spermatozoa had spent very little time in the epididymis and that their intracellular antioxidants have not been depleted. The increase in sperm viability towards the end of the DE period can probably be related to the decreased ROS levels as the spermatozoa would subsequently be less exposed to lipid peroxidation or apoptosis. These speculations can be partially supported by a negative correlation (r = −0.35, data not showed) that was found between ROS and viability. Likewise, the role of ROS in relation to normal sperm physiology and senescence must be addressed in future studies, specifically investigating the effect on capacitation and the acrosome reaction. It can therefore be deducted that prolonged sexual abstinence periods can have a detrimental effect on sperm function and could affect sperm quality [14]. Further studies are required to determine if low antioxidant activity and high oxidative stress in the epididymis itself are associated with poor fertility [16] and to determine the relation between abstinence and sperm conventional and functional characteristics.

The DNA integrity of spermatozoa has been considered as an important parameter in fertility studies [25, 26, 28, 42, 43]. Evenson et al., found that semen samples with a DFI of more than 29 % has an increased likelihood of reduced fertility [27]. The results from the present study showed that DFI remained below the suggested fertility threshold over the first 13 days of DE. Similar studies have shown that short abstinence periods lead to a greater reduction in the incidence of sperm DNA fragmentation and an increase on pregnancy rates after assisted reproductive techniques [11, 12]. During the whole process of spermatogenesis and maturation, the nuclear content gets condensed through a process whereby histones are replaced by protamines. This helps to protect the chromatin in the head of the spermatozoon. It is argued that an extended DE period (i.e. frequent ejaculations over a prolonged period of time) can lead to insufficient time for spermatozoa to mature. This will have a negative influence on the chromatin quality and can be associated with immature nuclear spermatozoa which are highly susceptible to DNA fragmentation. This can, in part, explain the rise to above the 29 % DFI threshold on the final day of the DE period.

To summarize, our results show that semen variables such as semen volume and total sperm count show a decrease when it is compared to the initial sample. However, parameters like progressive motility, morphology, MMP, DFI and plasma membrane integrity remain similar to the initial evaluation while others such as viability and ROS levels showed some improvement towards the end of the tested DE period.

This is the first study to report conventional and functional semen parameters over a 13 day period of frequent ejaculation. The results support our assumption that a DE period of two weeks does not influence seminal parameters negatively and can thus be used as an approach that can be applied during assisted reproductive technology, but within limitation as the data was collected from healthy controls and may not be applicable to males suffering from oligozoospermia and/or erectile dysfunction [11].

## Conclusions

In conclusion, our data suggests that frequent daily ejaculation of two weeks have no major negative effect on both conventional and functional parameters. The implication of this is extremely relevant clinically as it means that men diagnosed with cancer can collect and bank several semen samples in quick succession prior to onset of chemotherapy. Furthermore, frequent daily ejaculations can be utilized as a treatment option in cases of male infertility problems related to oxidative stress.

## Abbreviations

DCFH-DA: 2’,7’-dichlorofluorescein diacetate
DE: Daily ejaculation
DFI: DNA fragmentation index
DIOC6: 3,3′-dihexyloxacarbocyanine iodide
ESHRE: European Society of Human Reproduction and Embryology
NO: Nitric oxide
NOS: (reactive nitrogen species)
MFI: Mean Fluorescence Intensity
MMP: Mitochondrial membrane potential
PI: Propidium iodide
ROS: (reactive oxygen species)
SCSA: Sperm Chromatin Structure Assay
WHO: World Health Organization
